# Establishment of a Neonatal Natural Transmission Model for Cytomegalovirus Vaccine Development

**DOI:** 10.1093/infdis/jiag170

**Published:** 2026-03-23

**Authors:** Teeba Jihad, Xiaoyuan S Chi, Zhiqing He, Kwok Lee, Elie Needle, Jakob Loschko, Jessica Goymer, Helen Wang, Helene Boigard, Camille Robertson, Jennifer Obregon, Kasey Wilson, Isis Kanevsky, Paul Liberator, Yury V Matsuka, Mark R Walter, Kathrin U Jansen, Xinzhen Yang, Philip R Dormitzer, Kena A Swanson, Nicholas J Buchkovich

**Affiliations:** Vaccines, Pfizer Research and Development, Pfizer, Inc, Pearl River, NewYork, USA; Vaccines, Pfizer Research and Development, Pfizer, Inc, Pearl River, NewYork, USA; Vaccines, Pfizer Research and Development, Pfizer, Inc, Pearl River, NewYork, USA; Vaccines, Pfizer Research and Development, Pfizer, Inc, Pearl River, NewYork, USA; Vaccines, Pfizer Research and Development, Pfizer, Inc, Pearl River, NewYork, USA; Vaccines, Pfizer Research and Development, Pfizer, Inc, Pearl River, NewYork, USA; Vaccines, Pfizer Research and Development, Pfizer, Inc, Pearl River, NewYork, USA; Vaccines, Pfizer Research and Development, Pfizer, Inc, Pearl River, NewYork, USA; Vaccines, Pfizer Research and Development, Pfizer, Inc, Pearl River, NewYork, USA; Vaccines, Pfizer Research and Development, Pfizer, Inc, Pearl River, NewYork, USA; Vaccines, Pfizer Research and Development, Pfizer, Inc, Pearl River, NewYork, USA; Pfizer Research Biostatistics, Collegeville, Pennsylvania, USA; Vaccines, Pfizer Research and Development, Pfizer, Inc, Pearl River, NewYork, USA; Vaccines, Pfizer Research and Development, Pfizer, Inc, Pearl River, NewYork, USA; Vaccines, Pfizer Research and Development, Pfizer, Inc, Pearl River, NewYork, USA; Department of Microbiology, University of Alabama at Birmingham, Birmingham, Alabama, USA; Vaccines, Pfizer Research and Development, Pfizer, Inc, Pearl River, NewYork, USA; Vaccines, Pfizer Research and Development, Pfizer, Inc, Pearl River, NewYork, USA; Vaccines, Pfizer Research and Development, Pfizer, Inc, Pearl River, NewYork, USA; Vaccines, Pfizer Research and Development, Pfizer, Inc, Pearl River, NewYork, USA; Vaccines, Pfizer Research and Development, Pfizer, Inc, Pearl River, NewYork, USA

**Keywords:** cytomegalovirus, vaccine, rhesus macaque, RhCMV, neonate, natural transmission

## Abstract

A human cytomegalovirus (CMV) vaccine to prevent congenital disease is a public health priority. We previously demonstrated that vaccine-elicited rhesus CMV (RhCMV) neutralizing titers and T-cell responses comparable to natural infection failed to protect from RhCMV infection after experimental oral challenge. Consequently, we established a natural transmission model in which newborn rhesus macaques are cohoused with their RhCMV-positive mothers and immunized 5 times between 0 and 24 months with glycoprotein B (gB), pentamer, pp65, and in 1 group viral interleukin 10. While no significant differences were observed in infection rate between the vaccine and placebo groups at 40 weeks after birth, partial protection was observed at week 52 (83.3% infection in placebo, 42.1%–50% in vaccine recipients). Within 14 weeks of juvenile macaques transfer to group housing, all shed RhCMV. These results suggest that the neonatal RhCMV natural transmission model may recapitulate observations in humans immunized with recombinant gB and can be a useful tool for evaluating CMV vaccine candidates.

## Teaser

We present a neonatal rhesus macaque RhCMV transmission model, where newborns are cohoused with RhCMV-positive mothers before group housing. Evaluation of CMV vaccine candidates demonstrated partial protection at week 52, indicating the value of this model to predict transmission dynamics postvaccination.

Human cytomegalovirus (HCMV) is the most common congenital infection, and can cause intellectual impairment, blindness, and hearing loss. Developing a vaccine to prevent congenital CMV infection has been a long-standing public health priority, but no CMV vaccine is currently approved. Viral fusion glycoproteins are potential vaccine antigens and immunization with the CMV fusogen, glycoprotein B (gB) in its postfusion form adjuvanted with MF59 demonstrated partial, short-term protection of recently postpartum CMV-seronegative women (approximately 50% protection for 42 months) and of adolescent girls (43% protection for about 3 years) [1, 2]. This partial efficacy may be enhanced by the inclusion of additional CMV antigens.

The CMV pentameric glycoprotein complex (PC) is required for CMV entry into epithelial cells and is a target of potently neutralizing antibodies (nAb) [3, 4]. Another viral protein, pp65, is a major target for cytotoxic T-cell responses in natural infections [5–7]. Accordingly, pp65-2 (a rhesus CMV pp65 homologue), has shown some protective effects in a rhesus infection model and limited clinical trials [8, 9].

Viral interleukin 10 (vIL-10) is a CMV-encoded mimic of the potent immunomodulatory human cytokine, interleukin 10 (IL-10), that despite sharing only 27% sequence identity with its human homologue [10], hijacks IL-10 intracellular signal transduction pathways to ultimately manipulate and suppress host immune responses [11, 12]. A previous study demonstrated some success against transmission using rhesus macaque CMV (RhCMV) vIL-10 as a potential vaccine antigen, mutated to disrupt binding of vIL-10 to the intraleukin-10 receptor 1 to avoid wild-type immunosuppressive functions, which could negatively impact the intended immune response to vaccine antigens [13, 14]. Therefore, in addition to RhCMV postfusion gB, PC, and pp65, we added vIL-10M2 to the cocktail of prototype vaccine antigens to be assessed in our rhesus macaque natural transmission model.

We previously tested a protein subunit vaccine candidate consisting of RhCMV postfusion gB and PC in combination with DNA encoding pp65 in an oral RhCMV challenge model of adult seronegative rhesus macaque [15]. As RhCMV is ubiquitous in rhesus macaque [16], specific pathogen-free colonies for such experiments are maintained in rigorous isolation. The RhCMV oral challenge model was chosen because RhCMV and HCMV have high sequence homology; over 90% of RhCMV open reading frames have orthologous HCMV protein families [17, 18]. It also provides a route of exposure that mimics the usual route of human infection, increasing the chances that the animal challenge results may predict vaccine candidate performance in humans [19].

Three immunizations with the QS21-adjuvanted mixture of RhCMV postfusion gB and PC, along with electroporation of a plasmid encoding pp65, elicited neutralizing titers and T-cell responses as high as, or higher than, those observed after natural RhCMV infection [15]. However, immunization had no consistent impact on the onset, quantity, or duration of RhCMV detection by polymerase chain reaction (PCR) in blood or urine of the macaques after 5 oral challenges with 5 × 10^6^ plaque forming units (PFU).

We thus concluded that viral evasion of the vaccine-induced immune responses and/or the artificial nature of the challenge may have contributed to the failure to protect against any level of infection. Although the rhesus model bears similarities to human disease, it is not a disease model: conclusions about vaccine efficacy based on this challenge study may underestimate vaccine efficacy in humans. Immune evasion by CMV is not unprecedented, with latent carriers frequently reinfected by additional strains [20, 21]. Indeed, a high proportion of the approximately 250-kb CMV genome encodes proteins involved in immune evasion [22, 23]. Consequently, we considered whether experimental oral challenge of macaques might be more difficult to protect against than natural transmission, as is observed in humans.

Newborn rhesus macaques do not have detectable RhCMV infection, typically acquiring the virus from their environment within their first year [24]. High-level RhCMV shedding is common in the saliva and urine of postpartum rhesus macaque mothers [16]. To more closely model potential protection from natural exposure to CMV, we immunized infant macaques with prototype vaccines, housed them with their mothers, and observed them for potential protection against natural acquisition of circulating RhCMV.

## METHODS

Additional details on methods can be found in Supplementary Materials.

### Ethics Statement

All animal protocols were approved by the Institutional Animal Care and Use Committees at the New Iberia Research Center (NIRC) and Pfizer. Studies adhered to the Guide for the Care and Use of Laboratory Animals and the standards of the Association for Assessment and Accreditation of Laboratory Animal Care (Animal Assurance No. 000452). Humane end points and euthanasia followed the guidelines of the American Veterinary Medical Association.

### Vaccination

**Figure 1.**
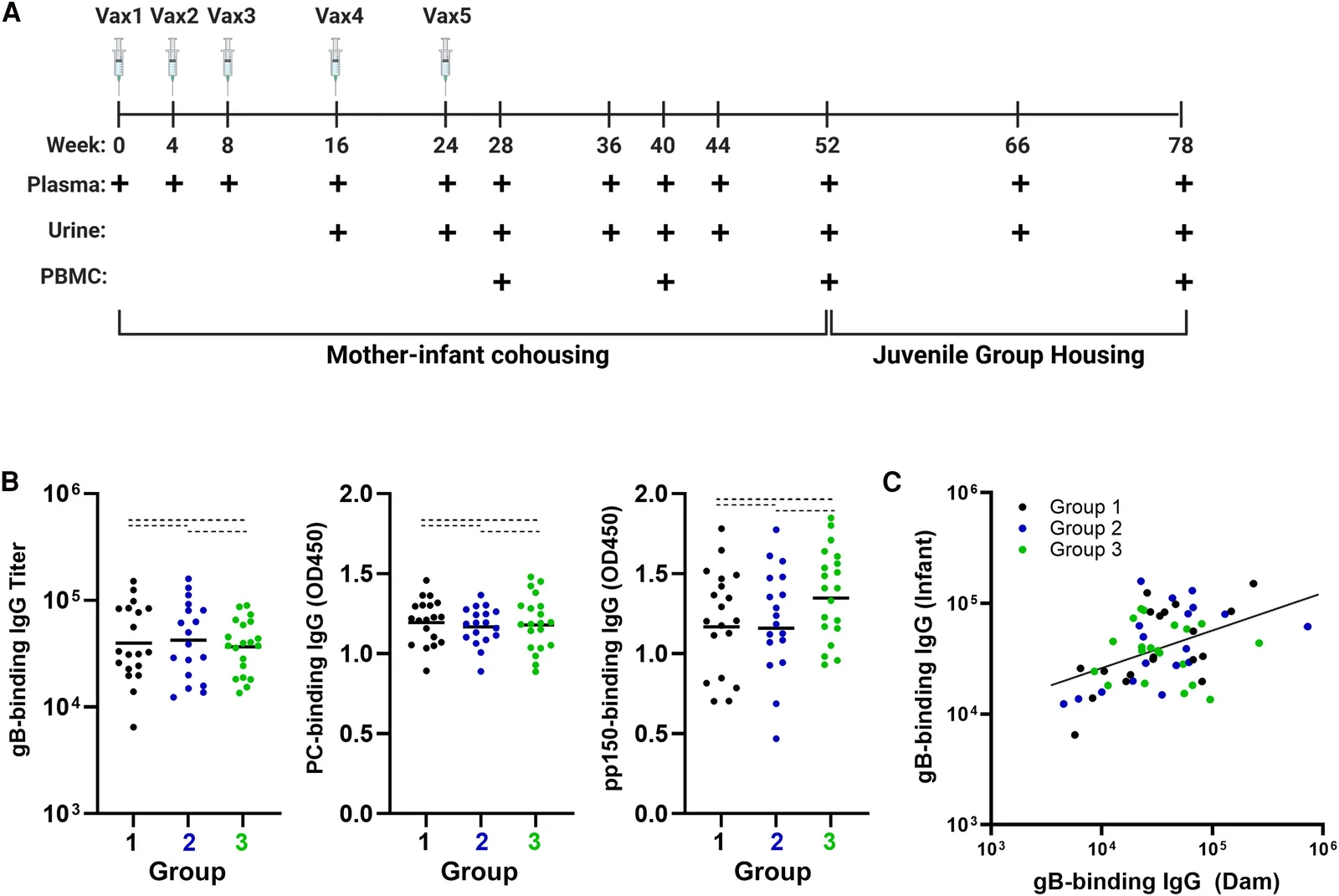
Study design and timeline and maternal-derived immunity. *A*, The 3 immunization groups consisted of group 1, QS-21 adjuvant in phosphate-buffered saline (placebo); group 2, 50 µg PC and 50 µg gB with QS-21, plus 0.2 mg pp65-2 DNA (PC/gB/pp65-2); and group 3, 50 µg PC, 50 µg gB674, and 45 µg vIL-10M2 with QS-21, plus 0.2 mg pp65-2 DNA (PC/gB/pp65-2/vIL-10M2). Various experimental procedures and their timing are indicated. Infants were cohoused with dams from birth to week 52, then released and housed with other infants (about 20 per crib). Created in BioRender (https://BioRender.com/xp6tj0h). *B*, Detection of anti-RhCMV gB-, PC-, and pp150-binding IgG in newborn macaques at week 0 prior to immunization. Plasma samples were serially diluted and tested against recombinant gB using a standard ELISA protocol as described in “Methods.” Each bar represents the geometric mean of titers from each group of macaques. No statistical difference between groups as determined by a 1-way ANOVA, *P* values were 0.853 for gB, .842 for PC, and .165 for pp150, dotted lines indicate *P* value > .05. *C*, Scatter plot of gB-binding titers of infants and respective dam. Line represents best fit calculated by nonlinear regression, log-log line analysis in Prism. Pearson *r* correlation coefficient was calculated on logged data, *r*^2^ = 0.447. Abbreviations: ELISA, enzyme-linked immunosorbent assay; gB, glycoprotein B; IgG, immunoglobulin G; OD450, optical density at 450 nm; PBMC, peripheral blood mononuclear cell; PC, pentamer complex; RhCMV, rhesus cytomegalovirus; Vax, vaccine; vIL-10M2, recombinant viral interleukin 10.

Rhesus macaques (*Macaca mulatta*) were bred and maintained at NIRC. Sixty newborns were assigned at birth to a vaccination group and cohoused with their mothers for 1 year before being weaned and moved to larger cribs with age-matched groups of approximately 20 animals. Immunization groups were as follows: 50 μg QS-21 only (group 1); 50 μg gB and 50 μg PC with 50 μg QS-21 and 0.2 mg pp65-encoding DNA (group 2); 50 µg gB, 50 μg PC, and 45 µg vIL-10M2 with 50 μg QS-21 and 0.2 mg pp65-encoding DNA (group 3). Dosages were chosen in accordance with previous nonhuman primate (NHP) studies and clinical CMV vaccine candidates [1, 15]. Protein antigens were delivered intramuscularly (IM) in a volume of 0.5 mL, and the pp65-2 DNA delivered IM in a 0.1-mL volume by electroporation using the TDS-IM system (Ichor Medical Systems). Animals were immunized within 3 days of birth, and boosted at weeks 4, 8, 16, and 24 (Figure 1).

### RhCMV Neutralization

Heat-inactivated plasma samples serially diluted were mixed with approximately 500 PFU/well of RhCMV UCD52 and added to monkey kidney epithelial (MKE) or Telo-RF cells. After 48 hours, cells were fixed and stained with a rabbit anti-RhCMV IE1 polyclonal serum and Alexa Fluor 488 goat anti-rabbit immunoglobulin G (IgG; Life Technologies). IE1-positive cells were counted using a CTL-ImmunoSpot S6 Analyzer and 50% neutralizing titers were calculated. Pooled plasma from CMV-positive macaques served as a positive control.

### Enzyme-Linked Immunosorbent Assay (ELISA)

A standard ELISA was used to detect plasma antibodies to viral proteins. Three-fold serial dilutions of sera starting at 1:100 were added to coated microplates (Immulon 2HB; ThermoFisher). Following addition of peroxidase-conjugated goat anti-monkey IgG (Southern Biotech), and a peroxidase 3,3',5,5'-tetramethylbenzidine (TMB) substrate (ThermoFisher), absorbance was recorded spectrophotometrically at a wavelength of 450 nm. The dilution was plotted against optical density at 450 nm using SoftMax and the value corresponding to 0.1 was taken as the titer.

### ELISPOT assay

Rhesus peripheral blood mononuclear cells (PBMCs) were collected from whole blood by gradient centrifugation using Ficoll-Paque Premium (Cytiva) and used fresh for ELISPOT assays according to the manufacturer's protocol (Mabtech). For stimulation, peptide pools spanning the open reading frames of pp65 and IE1 (15mers with 11 amino acid overlap) were designed and ordered. ELISPOT plates were developed according to Mabtech's NHP IFNγ ELISPOT protocol using 3-amino-9-ethylcarbazole (AEC) substrate (Vector Labs). Plates were scanned with an ImmunoSpot Analyzer, and spots were counted with ImmunoSpot Software (Cellular Technology, Ltd), with limit of detection (LOD) set to 1 spot forming cell/10^5^ PBMCs.

### vIL-10 Inhibition

To measure interferon-γ (IFN-γ) secretion in lipopolysaccharide (LPS)-stimulated human PBMCs (StemCell) treated with vIL-10M2, heat-inactivated sera was diluted and mixed with 5 µg/mL LPS (Sigma) with and without 1 ng/mL of vIL-10M2 prior to addition. Cytokine release was analyzed by a Luminex assay using ProcartaPlex Immunoassay kit following manufacturer instructions (ThermoFisher). The percentage of inhibition is the relative concentration of IFN-γ released in the presence of the heat-inactivated NHP sera with vIL-10M2 over the concentration of IFN-γ released in the presence of plasma without vIL-10M2.

### qPCR detection of viral load

DNA was extracted from 400 µL of urine samples using a QIAamp DSP Virus/Pathogen Midi kit (QIAGEN) according to manufacturer's instructions. Real-time quantitative PCR (qPCR) assay was performed using primers located in a conserved region of the RhCMV gB gene [15]. Positive samples were defined as > 10 genome copies.

## RESULTS

### Immunization of Newborn Macaques

To study the effects of 2 CMV vaccine candidates containing PC, gB and pp65, either with or without vIL-10M2, 60 pregnant rhesus macaques were enrolled whose newborns were randomly assigned to 1 of 3 study groups: placebo (group 1), PC/gB/pp65-2 (group 2), or PC/gB/pp65-2/vIL-10M2 (group 3). Each newborn macaque received the first intramuscular immunization within 3 days of birth, and a total of 5 vaccinations were given through week 24 (Figure 1*A*). PC, gB, and vIL-10M2 were administered as subunits while pp65 was administered as electroporated DNA. Three subjects were excluded during the study due to health issues. Blood (for serum and PBMCs) and urine were collected at defined intervals throughout the study (Figure 1*A*). All newborn macaques had anti-gB, anti-PC, and anti-pp150 antibodies, presumably acquired by transplacental transfer from their CMV-seropositive mothers (Figure 1*B*). The overall Pearson *r* correlation coefficient showed a weak positive correlation between levels of maternal and infant antibodies (Figure 1*C* and Table 1). There was no significant difference in the initial neutralizing titers among the groups. To monitor infection status, beginning at week 16 and continuing at select time points (Figure 1*A*), genome copies were quantified from urine by qPCR. Over time, a subset of infant macaques acquired RhCMV from their cohoused mothers. At the initial 16-week time point, 7 infants (12.3%) tested positive for RhCMV. By 24 weeks of age, corresponding to the last vaccine dose, 19 out of 57 infants (33.3%) were infected, with 5 (26%), 6 (33%), and 8 (40%) infants from groups 1, 2, and 3, respectively, testing positive (Supplementary Figure 1). The increase in infected monkeys from week 16 to week 24 demonstrates evidence of CMV natural transmission.

**Table 1.** Individual Mother-Infant Rhesus Macaque Pairs’ gB-Binding IgG Titers Organized by Vaccination Group

Group 1			Group 2			Group 3		
Mother-Baby Pair No.	Mother gB IgG Titer	Baby gB IgG Titer	Mother-Baby Pair No.	Mother gB IgG Titer	Baby gB IgG Titer	Mother-Baby Pair No.	Mother gB IgG Titer	Baby gB IgG Titer
1-1	82 185	33 244	2-1	729 444	61 648	3-1	24 491	18 902
1-2	29 332	32 699	2-2	21 894	62 774	3-2	80 225	65 521
1-3	8276	13 934	2-3	60 480	80 580	3-3	23 066	40 359
1-4	10 544	24 397	2-4	22 659	158 654	3-4	95 785	13 513
1-5	29 157	31 345	2-5	35 102	14 922	3-5	45 553	63 439
1-6	67 207	30 954	2-6	47 237	27 556	3-6	27 762	39 711
1-7	148 662	84 613	2-7	58 288	38 900	3-7	66 274	18 234
1-8	236 219	151 121	2-8	61 352	29 313	3-8	265 598	43 826
1-9	18 183	22 668	2-9	130 276	80 114	3-9	55 763	15 353
1-10	5730	6486	2-10	67 098	91 735	3-10	54 361	28 278
1-11	6429	25 863	2-11	43 546	111 561	3-11	58 963	58 766
1-12	37 061	83 352	2-12	4530	12 334	3-12	33 329	35 763
1-13	27 591	83 698	2-13	6163	13 717	3-13	23 072	37 406
1-14	25 778	124 655	2-14	65 983	130 628	3-14	12 642	45 356
1-15	80 973	19 692	2-15	23 783	49 854	3-15	24 099	86 676
1-16	16 629	19 731	2-16	25 012	28 887	3-16	8593	24 311
1-17	33 561	76 937	2-17	19 116	19 893	3-17	19 304	73 476
1-18	67 374	56 108	2-18	10 006	15 786	3-18	11 419	18 123
1-19	46 610	98 223	…	…	…	3-19	32 142	37 434
…	…	…	…	…	…	3-20	22 719	89 160

Abbreviations: gB, glycoprotein B; IgG, immunoglobulin G.

### IgG Levels Increase in Vaccinated Macaques, but Decline in the Placebo Group, During the First 12 Months of Life

**Figure 2.**
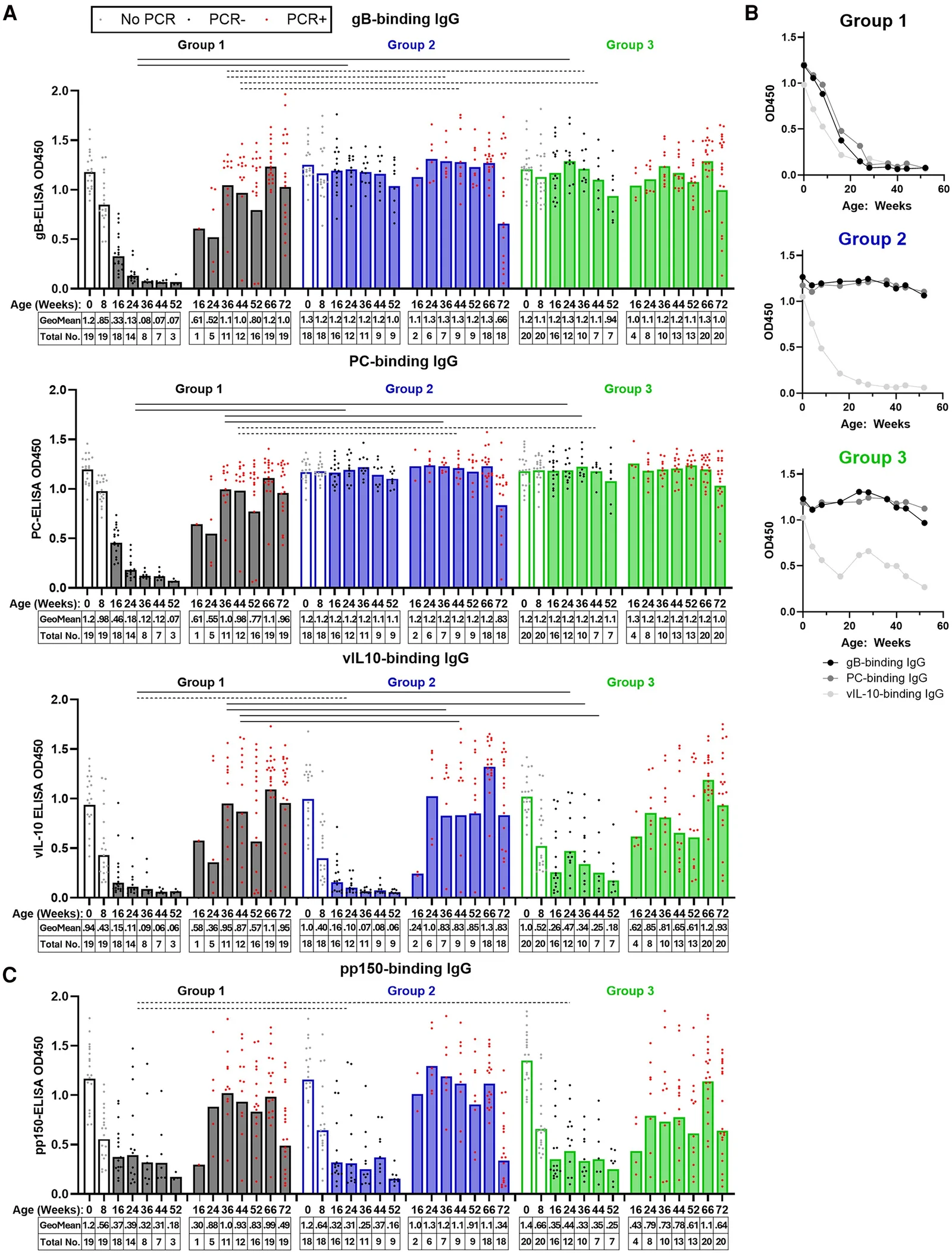
IgG response to vaccination and infection. *A*, Kinetics of IgG against vaccine antigens gB, PC, and vIL-10M2. Data are separated by vaccination group and infection status, measured by viral shedding in urine as detected by PCR. Each bar represents the geometric mean of OD450 values by ELISA and each dot represents data from an individual animal. For gB-binding IgG at week 24, titers in vaccinated, uninfected infants were significantly higher than placebo uninfected animals (*P* ≤ .001). At week 36 and 44, no statistical difference was observed between infected animals in group 1 and infected animals in group 2 or group 3 (*P* = .429 and *P* = .299). For PC-binding IgG at week 24, titers in vaccinated, uninfected infants were significantly higher than placebo, uninfected animals (*P* ≤ .001). For IgG at week 44, no statistical difference was observed between infected animals in group 1 and infected animals in group 2 (*P* = .581) or uninfected animals in group 3 (*P* = .516). For vIL10-binding IgG at week 24, no statistical difference was observed between uninfected animals in group 1 and uninfected animals in group 2 (*P* = .952). Animals in group 3 had statistically higher vIL10-binding IgG compared to animals in the control group (*P* ≤ .001), but these levels were significantly lower than those postinfection (*P* = .001). For pp150-binding IgG at week 24, no significant difference was observed between uninfected animals in group 1 versus uninfected animals in group 2 or group 3. *B*, Data from PCR-negative animals (< 10 copies of virus) in (*A*) regraphed by vaccination group to show durability of maternal-derived and vaccine-induced IgG response. *C*, Kinetics of IgG against nonvaccine antigen pp150 as depicted for vaccine antigens in (*A*). *A* and C, Solid lines indicate *P* value < .05 and dotted lines indicate *P* value > .05 as determined by a 1-way ANOVA with group followed by Dunnett comparisons with group 1 as the reference. Abbreviations: ELISA, enzyme-linked immunosorbent assay; gB, glycoprotein B; GeoMean, geometric mean; IgG, immunoglobulin G; OD450, optical density at 450 nm; PC, pentameric glycoprotein complex; PCR, polymerase chain reaction; vIL-10M2, recombinant viral interleukin 10.

In the placebo group (group 1), the IgG titers against vaccine antigens steadily waned over the first 6 months of life, representing the decline of passively acquired maternal antibodies (Figure 2*A* and 2*B*), in agreement with a previous study [16]. In the vaccinated groups, IgG titers against gB and PC were significantly higher than in unvaccinated animals and were maintained at high levels during the first 12 months (Figure 2*A* and 2*B*), regardless of infection status. Notably, at weeks 36 and 44, similar IgG titers against gB were observed in unvaccinated, infected macaques compared to vaccinated, uninfected animals, demonstrating that vaccination in this model could achieve the same level of antibodies as infection. Similarly, IgG-binding titers against PC after vaccination were at or above levels observed after infection alone at weeks 36 and 44. In group 3 vaccinated animals, IgG titers against vIL-10 were significantly higher than in the adjuvant-only placebo group at 24 weeks with titers declining over time but still remaining higher than in uninfected animals not vaccinated with vIL-10M2. Unlike for gB and PC, vIL-10M2 vaccination induced significantly lower IgG titers against vIL-10 than those elicited after infection alone. IgG titers for pp150, a nonvaccine antigen, declined during the first 6 months and only rebounded upon infection (Figure 2*C*). These findings indicate both vaccines produced IgG responses that remained higher than those seen after natural infection.

### Virus Neutralizing Antibody Responses

**Figure 3.**
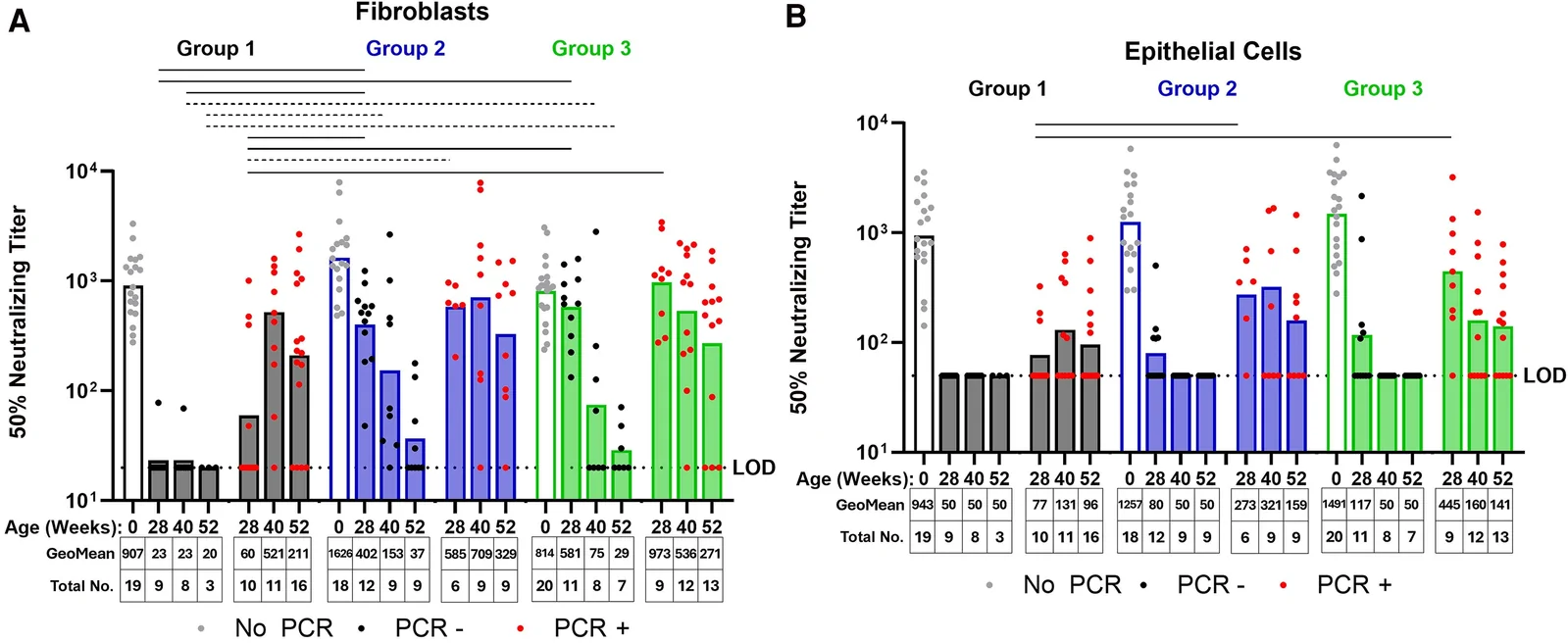
Virus neutralization following vaccination and infection. The nAb titer of individual samples was determined using UCD52 virus in (*A*) fibroblasts (Telo-RF) and (*B*) epihelial cells (primary MKE cells) as described in “Methods.” The limit of detection of 50% neutralizing titer was 50 and 20 for the MKE cell assay and Telo-RF cell assay, respectively. Samples that did not generate a positive neutralization titer were assigned a value of 20 for Telo-RF cell assay and 50 for MKE cell assay. Each bar represents the geometric mean of ED_50_ from each group of macaques, and dots represent data from individual animals. Data are presented by vaccination group and infection status. *A* and *B*, Solid lines indicate *P* value < .05 and dotted lines indicate *P* value > .05 as determined by a Kruskal-Wallis test with Dunn multiple comparisons or a 1-way ANOVA followed by Dunnett comparisons with group 1 as the reference. In fibroblasts at week 28, no significant difference was observed between infected animals in group 1 and infected animals in group 2 (*P* = .060). At week 40, no statistical difference was observed between group 1 and group 3 uninfected animals (*P* = .182). At week 52, there was no statistical difference between uninfected animals in group 1 compared to group 2 (*P* = .403) or group 3 (*P* = .403). Abbreviations: ED_50_, 50% effective dose; GeoMean, geometric mean; LOD, limit of detection; MKE, monkey kidney epithelial cell; nAb, neutralizing antibody; PCR, polymerase chain reaction.

As CMV infection occurs in multiple cell types, with different surface glycoproteins required for entry [25], virus neutralization assays were performed in both fibroblasts (Telo-RF) and epithelial cells (MKE). Most macaques in groups 2 and 3 developed nAb titers after vaccination, as shown in fibroblasts (Figure 3), with both groups showing higher neutralization titers than placebo at week 28. In PCR-negative animals, these titers decreased over time, and by week 52 neither group differed significantly from placebo. Neutralizing titers measured on fibroblasts did not indicate protection from infection, as previously demonstrated [15]. At weeks 28 and 40, 1 macaque in group 1 (placebo) had fibroblast nAb in the absence of infection, likely a result of persisting maternal antibodies.

In contrast, epithelial cell neutralization titers following either natural acquisition of infection or vaccination failed to reach the levels comparable to maternal nAbs at week 0 (Figure 3). At week 28, epithelial cell nAb titers in both vaccine groups were similar regardless of infection status. After week 28, vaccinated animals with PCR-confirmed infection demonstrated substantial boosting of nAb titers. In contrast, nAb titers continued to wane through week 52 in macaques that remained uninfected. Vaccinated, infected animals in group 3 had significantly higher nAbs compared to infected animals in the placebo group. A similar trend was observed for group 2, although it did not reach statistical significance. As observed in both epithelial cells and more prominently in fibroblasts, neutralizing titers waned over time regardless of infection status. These results confirm that immunization of infant macaques results in the production of nAb to similar or higher levels than observed following natural infection.

### vIL-10 Vaccination Elicits IL-10 Inhibitory Antibodies

**Figure 4.**
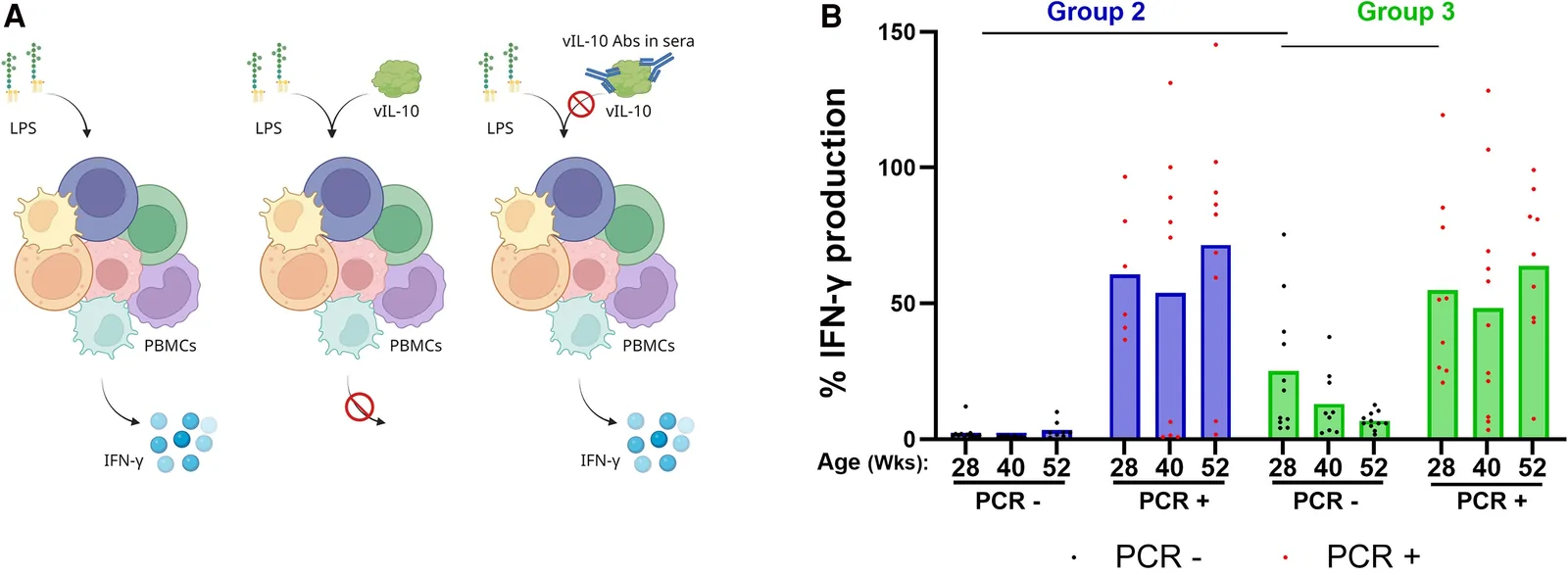
Functional effect of vIL-10M2 vaccination. *A*, Schematic representing vIL-10 inhibition assay. Created in BioRender (https://BioRender.com/e81de39). *B*, Percent inhibition of vIL-10 activity in animals receiving vaccines with and without vIL-10M2 (group 2 and group 3). A 1:400 dilution of plasma samples collected at the indicated times postvaccination was added to the PBMCs in media containing LPS with and without vIL-10M2. After overnight incubation, IFN-γ secretion from the LPS-stimulated human PBMCs was measured by a Luminex assay and the percentage IFN-γ produced was calculated by comparison of relative IFN-γ secretion in the presence of sera from vaccinated animals versus unvaccinated, uninfected animals, as described in “Methods.” Solid line indicates *P* value < .05 as determined by 2-sided 2-sample *t* test. Abbreviations: Ab, antibody; IFN-γ, interferon-γ; LPS, lipopolysaccharide; PBMC, peripheral blood mononuclear cell; PCR, polymerase chain reaction; vIL-10M2, recombinant viral interleukin 10.

Because vIL-10 is not required for viral entry, antibodies against vIL-10 would not be expected to neutralize virus but rather block the immune suppressor activity of vIL-10. vIL-10 is utilized by the virus to suppress detection of infected cells by the immune system through various mechanisms [26]. Thus, to evaluate whether vIL-10M2 immunization elicited antibodies that could functionally block the immune suppressor activity of vIL-10, a Luminex assay was developed that takes advantage of the ability of vIL-10 to block IFN-γ secretion from human PBMCs when stimulated with bacterial LPS (Figure 4*A* and Supplementary Figure 2) [27]. Plasma samples from the vaccinated monkeys of group 2 (without vIL-10M2) and group 3 (with vIL-10M2) at 3 postvaccination time points were analyzed. All animals that shed virus had high anti–vIL-10 inhibiting antibodies. In uninfected animals, those in group 3 had significantly higher anti–vIL-10 IgG levels and percent vIL-10 inhibition compared with those in group 2, suggesting a specific response against the vIL-10M2 vaccine antigen (Figure 4*B*). IFN-γ production was significantly lower in uninfected, vaccinated animals of group 3 when compared to the infected animals within this group, suggesting vaccination was not as effective as infection at eliciting functional vIL-10 antibodies. Furthermore, in the absence of infection-elicited immunity, the ability of group 3 animals’ sera to inhibit vIL-10 decreased dramatically in the 6 months following vaccination, corresponding to a reduction in vIL-10–binding IgG levels. These results suggest that although functional antibodies against vIL-10 were generated following immunization, in this model, vIL-10 antibody levels did not reach those following natural infection and quickly waned.

### pp65-2 DNA Vaccination Elicits Cellular Immune Responses

**Figure 5.**
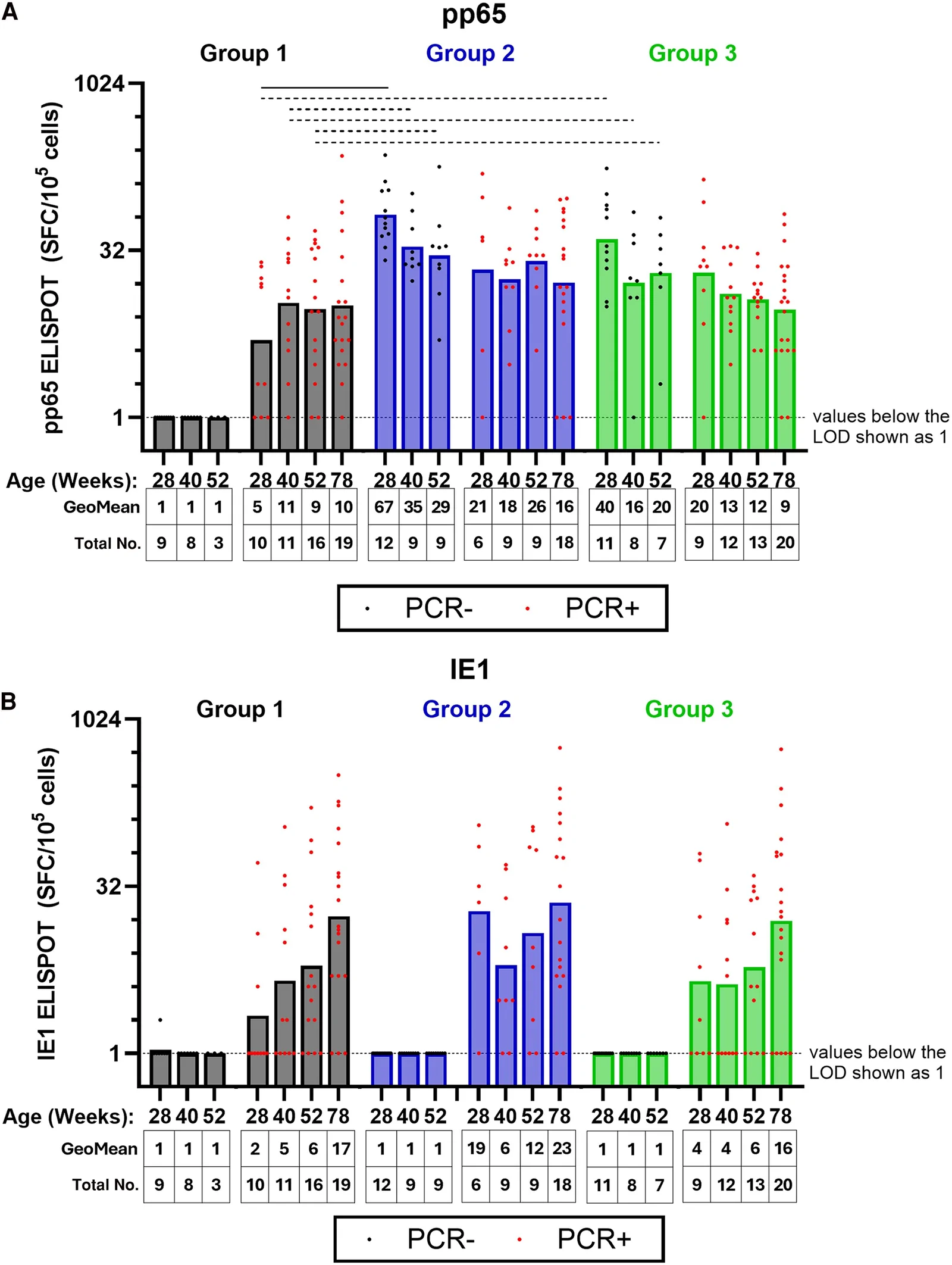
Cellular immune response to vaccination and infection. Frequency of RhCMV pp65 (*A*) or IE1-specific (*B*) IFN-γ–producing cells was determined by ELISPOT in unvaccinated controls and vaccinated animals at week 28, 40, 52, and 78 weeks as described in the “Methods.” Data are plotted by vaccination group and infection status. Values below the LOD are shown as 1. Placebo group, n = 19; PC + gB + pp65 group, n = 18; PC + gB + pp65 + vIL-10 group, n = 20. *A*, Solid lines indicate *P* value < .05 and dotted lines indicate *P* value > .05 as determined by a 1-way ANOVA with group followed by Tukey multiple comparisons. For pp65 at week 28, no significant difference was observed between infected animals in group 1 and uninfected animals in group 3 (*P* = .062). For pp65 at week 40, no significant difference was observed between infected animals in group 1 and uninfected animals in group 2 (*P* = .104) or group 3 (*P* = .785), nor at week 52 (*P* = .117 and .723, respectively). Abbreviations: gB, glycoprotein B; GeoMean, geometric mean; IFN-γ, interferon-γ; LOD, limit of detection; PC, pentameric glycoprotein complex; PCR, polymerase chain reaction; RhCMV, rhesus cytomegalovirus; SFC, spot-forming cell.

To evaluate the RhCMV pp65-2 antigen-specific CD8^+^ T-cell responses, PBMCs from weeks 28, 40, 52, and 78 were analyzed by IFN-γ ELISPOT (Figure 5*A*). Four weeks postimmunization, most vaccinated animals had detectable pp65-2–specific CD8^+^ responses, regardless of infection status. In contrast, pp65-2–specific CD8^+^ T-cell responses were only present in the placebo group in animals that had acquired a RhCMV infection. At week 28, the pp65-2–specific CD8^+^ T-cell response in group 2 and group 3 uninfected, vaccinated animals was significantly higher than those observed for infected, placebo animals, with mean differences of 71.1 and 47.1, respectively. In the vaccinated groups (groups 2 and 3), the pp65-2-specific CD8^+^ T-cell responses were maintained throughout the first year of life, even in uninfected animals, remaining at or above levels observed following infection. As a control, CD8^+^ T-cell responses against a nonvaccine antigen, IE1 (i.e animals would only have been exposed to this antigen through infection), were investigated. In comparison to vaccine antigens, IE responses were only observed in PCR-positive animals (Figure 5*B*). Together, these data demonstrate that vaccination with pp65-2–encoding DNA produces a sustained pp65-2–specific CD8^+^ T-cell response in infant macaques.

### Vaccinated Macaques Exhibit Reduced Viral Infection at Week 52

**Figure 6.**
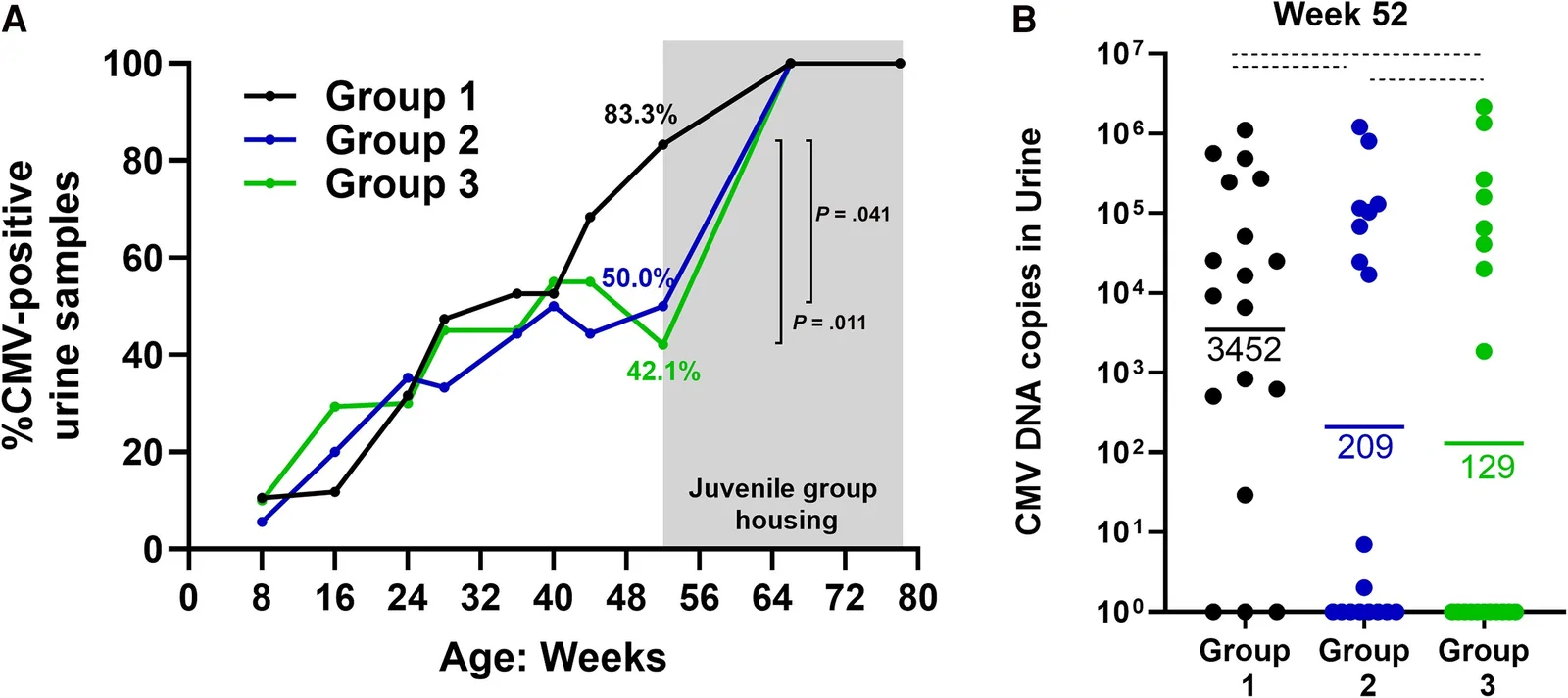
Effect of vaccination of infant macaques on CMV infection status. *A*, Virus shedding was measured in DNA extracted from urine using qPCR. A Ct value of 40 was deemed as negative. Y-axis indicates the percentage of PCR positive in the evaluable animals in each group, and x-axis shows the time points of sample collection in experiment weeks. In group 2, one sample was not available for evaluation. Statistical analysis was performed as described in the “Methods” section. *B*, CMV DNA copies in urine as measured by PCR for each animal at 52 weeks postvaccination. The geometric mean is depicted by a bar with corresponding value. Zero values are set to 1 for plotting on logarithmic axis. Dotted line indicates *P* value > .05 as determined by a nonparametric Kruskal-Wallis test. Abbreviations: CMV, cytomegalovirus; Ct, cycle threshold value; qPCR, quantitative polymerase chain reaction.

Although infant macaques in all 3 groups became infected at similar rates through week 40 of life, by week 44 a lower proportion of vaccinated macaques shed RhCMV relative to the placebo group. Fisher exact test indicated these differences reached statistical significance beginning at week 52, where the percentages of positive urine samples were 83.3%, 50%, and 42.1%, for group 1, group 2, and group 3, respectively (Figure 6*A*). The addition of vIL-10M2 did not significantly enhance protection. A second method to confirm infection is to assess T-cell activity against a nonvaccine antigen. The lower infection rates in the vaccinated groups correlated with lower positivity in ELISPOT of the nonvaccine antigen RhCMV IE1 (Figure 5). Odds ratios revealed that monkeys in the placebo, group 1 were 4.8- and 6.5-times more likely to be infected than monkeys in groups 2 and 3, respectively. Although the geometric mean viral burden for the vaccinated groups trended lower, the difference between vaccinated and unvaccinated was not statistically significant (Figure 6*B*). Together, these data demonstrate that both vaccine candidates reduced the incidence of viral shedding within 1 year postvaccination. However, after juvenile macaques were separated from their mothers at 52 weeks and moved to a larger crib with approximately 20 age-matched animals, by week 66, all animals were positive for CMV infection by PCR, regardless of vaccine group (Figure 6*A*). There were no significant difference in the viral loads in monkeys in each crib at the time of assignment (Supplementary Figure 3).

## DISCUSSION

The rhesus macaque model is an important tool for evaluating CMV vaccine candidates [28, 29]. However, the small number, low availability, and high cost of CMV-free, adult macaques is a limitation to effective preclinical evaluation of CMV vaccine efficacy. Most rhesus infants are exposed to CMV during their first year of life [30], making them a potential candidate for natural acquisition studies for more meaningful assessments of CMV vaccine efficacy, although the suitability of infant macaques for this purpose was unknown. We demonstrated that newborn macaques are indeed a suitable model to assess the protective efficacy of CMV vaccine candidates against natural RhCMV transmission as we evaluated transmission, persistence, and shedding of RhCMV in infant macaques exposed to infected cage mates. All newborns acquired anti-RhCMV antibodies (likely by transplacental transfer), which diminished approximately 6 months after birth in the absence of active infant RhCMV infection or vaccination. The rate of viral acquisition and shedding was similar in all vaccine and placebo groups through week 40, despite the detection of immune responses against vaccine antigens in all vaccinated animals. Of note, neutralization responses were higher in infected monkeys that were vaccinated, which may indicate that vaccination enhances neutralization responses to a subsequent infection, although the importance of these nAbs with respect to protection from infection remains unclear. Between weeks 44 and 52, an increasing proportion of placebo recipients shed virus whereas the proportion of vaccine recipients that shed virus plateaued. This is the first demonstration in a NHP model of partial vaccine efficacy against virus shedding following CMV horizontal infection, providing evidence that neonatal NHPs are valuable for evaluating CMV vaccine candidates under more natural conditions.

HCMV vaccine candidates that include PC, gB, and pp65-2 have been shown to elicit both humoral and cellular immune responses [31, 32]. However, our earlier studies with specific pathogen-free adult rhesus macaques show that homologous CMV equivalents of these antigens also elicit immune responses but do not prevent viral transmission, despite the presence of robust RhCMV nAb [15]. CMV encodes numerous immune-regulating gene products to evade the host immune system [23]. Among them, vIL-10 is reported to be a key regulator of host immune responses to RhCMV, as vaccination with recombinant vIL-10 proteins (vIL-10M1 and vIL-10M2) reduced viral transmission [14]. In this study, maternally derived serum anti-vIL-10 IgG levels in unvaccinated and uninfected infants declined rapidly over the first 6 months of life. A similar decline was also seen previously in specific pathogen-free macaques immunized with vIL-10M2 DNA or proteins [33]. Animals vaccinated with vIL-10M2 (group 3), showed anti–vIL-10 IgG responses, although at lower levels than those of infected animals. During the first year, there were no significant differences in the proportion of animals shedding virus when comparing the vaccinated groups with and without vIL-10M2, suggesting vIL-10M2 did not provide an efficacy benefit in this model.

One major strength of this study is the use of a natural RhCMV transmission model that more closely resembles HCMV transmission among humans than traditional experimental inoculation models. Variables to consider in this transmission model include unequal exposures to different virus strains and quantities between animals. At 1 year of age, all macaques were released from cohousing with their mothers, and moved to corn cribs (outside enclosures), each of which housed approximately 20 randomly assigned mixed-group similarly aged animals. The number of macaques shedding virus at week 52 when assigned to group housing were 10, 12, and 9 among groups 1 (placebo), 2 (vaccine without vIL-10M2), and 3 (vaccine with vIL-10M2), respectively. No significant difference was observed in the average number of collective genomes being shed by the 3 groups. A follow-up sampling 14 weeks later (week 66) showed all animals were infected, potentially because of higher exposure and more diversity of RhCMV circulating in the environment. While this indicates that vaccination only afforded delayed protection from infection, this transmission model is more faithful to real-world scenarios where children are exposed to various levels of virus in a natural setting via group care or through infected home contacts, and as such provides valuable additional insight into potential vaccination strategies. We cannot make any conclusions from our study on the impact of maternal immunity on the vaccine response by newborn macaques [34]. Even so, our vaccine was given multiple times from birth to 6 months, and IgG titers in the immunized and nonshedding animals were maintained at high levels through 12 months of age. The effect of breast milk on infection and immunity was not assessed, although it could also be investigated in the context of this model. While our study demonstrates that a vaccine candidate that elicits both humoral and cellular immune responses against CMV antigens was not protective, this study demonstrates that the natural transmission neonate model is a valuable tool for future evaluation of CMV vaccine candidates [35–38].

## Supplementary Material

jiag170_Supplementary_Data

## Supplementary Data

Supplementary materials are available at *The Journal of Infectious Diseases* online (http://jid.oxfordjournals.org/). Supplementary materials consist of data provided by the author that are published to benefit the reader. The posted materials are not copyedited. The contents of all supplementary data are the sole responsibility of the authors. Questions or messages regarding errors should be addressed to the author.
