# Supplementary material for: Establishment of a Neonatal Natural Transmission Model for Cytomegalovirus Vaccine Development
Source: J Infect Dis. 2026 Mar 23;234(3):e583–94. doi: 10.1093/infdis/jiag170 (PMC13600383; doi:10.1093/infdis/jiag170)
Supplement: Supplementary file 1 [file jiag170_supplementary_data.docx]

**Supporting Methods**

*Production of vaccine immunogens*

The construction, expression, and purification of a soluble version of RhCMV strain UCD52 pentamer complex (HQ667932, HQ667933, GU552456) and post-fusion gB ectodomain (ADD92468) lacking the transmembrane and cytoplasmic regions with a C-terminal histidine tag, and generation of the plasmid encoding pp65-2 (QQL11309) has been described previously [[1](bookmark://_ENREF_1)]. The RhCMV vIL-10M2 antigen (AF202536.1) was produced from protein expressed in Drosophila Schneider S2 cells and purified by affinity chromatography as previously described [[2](bookmark://_ENREF_2)]. Wild-type vIL-10 (AAF59907.1) was purified from Chinese hamster ovary cells using a C-terminal strep tag and a StrepTrap HP column (GE Healthcare).

*Virus and cells*

RhCMV strain UCD52, primary monkey kidney epithelial cells (MKE) and telomerized rhesus fibroblast cells (Telo-RF) were kindly provided by Dr. Peter Barry (University of California, Davis) [1-3]. Virus stocks were titrated on MKE or Telo-RF cells using a single-round entry assay enumerating infected cells by immediate-early-1 (IE1) immunostaining 48-hours after virus inoculation [4].

*Sample Collection*

Vaccination and sample collection are outlined in Figure 1. Blood in heparinized tubes for plasma and peripheral blood mononuclear cells (PBMCs), and urine by disposable catch pan, were collected as indicated. No urine sample was obtained for one macaque in Group 2 at week 52. One animal from Group 1 and two animals from Group 2 were excluded from the study due to dehydration, resulting in euthanasia or death.

*Statistical Methods*

Statistical analyses were performed in R (version 4.4.1). Both parametric and non-parametric methods were used to analyze response data, with all tests evaluated at a significance level of α = 0.05. Parametric approaches were applied when model assumptions were met, and some responses were log-transformed to improve normality. When assumptions were violated, particularly where many observations were at LOD, resulting in highly skewed distributions with minimal variability, non-parametric methods were used. Specific tests and detailed analyses are detailed as follows:

- In vitro RhCMV neutralization assay; statistical analysis of 50% neutralization titers was performed using a Kruskal-Wallis test followed by Dunn’s multiple comparisons or a one-way ANOVA followed up with Dunnett’s comparisons with Group 1 as the reference.
- ELISA detection of anti-RhCMV antibodies; statistical analysis on IgG-binding titers included a one-way ANOVA followed by Dunnett’s comparisons with Group 1 as the reference. To evaluate the relationship between maternal and infant gB-binding IgG, a Pearson r correlation coefficient was calculated.
- ELISPOT; statistical analysis of spots was performed using a one-way ANOVA followed by Tukey’s Multiple Comparisons test.
- vIL-10 inhibition assay; statistical analysis of percent INF-γ inhibition was calculated using a two-sided two-sample t-test.
- qPCR; statistical tests applied to viral genome copies or number of monkeys infected include a non-parametric Kruskal-Wallis test and Fisher’s exact test.

**Supporting Information**

**Figure S1. Infection status at 24 weeks post infection.** Number of viral genomes detected in individuals from each vaccination group at 24 weeks post infection. The geometric mean is represented by a bar and the LOD is denoted by a dotted line with all PCR negative animals graphed at the level of detection. Dotted line indicates p-value >0.05 as determined by a non-parametric Kruskal-Wallis test.

**Figure S2. Dose response of wild-type RhCMV vIL-10 inhibition**. IFN-γ secretion from human PBMCs stimulated by 5, 7.5 or 10 μg/ml of LPS in the presence of no vIL-10 or 0.5, 1, 2.5 and 5 ng/ml of vIL-10. Bars represent mean values of 4 independent samples, represented by dots.

**Figure S3. Infection status at 52 weeks post infection.** Number of viral genomes detected in individuals assigned to the three congregate housing groups. The geometric mean is represented by a bar and the LOD is denoted by a dotted line with all PCR negative animals graphed at the level of detection. All 0 values were converted to 1 to be graphed on log scale. Dotted line indicates p-value >0.05 as determined by a non-parametric Kruskal-Wallis test.

**Figure S1**

**Figure S2**

**Figure S3**.

**References for Supporting Information**

1.	Chang WL, Kirchoff V, Pari GS, Barry PA. Replication of rhesus cytomegalovirus in life-expanded rhesus fibroblasts expressing human telomerase. J Virol Methods. 2002;104(2):135-46. Epub 2002/06/29. doi: 10.1016/s0166-0934(02)00060-5. PubMed PMID: 12088823.

2.	Oxford KL, Strelow L, Yue Y, Chang WL, Schmidt KA, Diamond DJ, et al. Open reading frames carried on UL/b' are implicated in shedding and horizontal transmission of rhesus cytomegalovirus in rhesus monkeys. J Virol. 2011;85(10):5105-14. Epub 2011/03/11. doi: 10.1128/jvi.02631-10. PubMed PMID: 21389128; PubMed Central PMCID: PMCPMC3126184.

3.	Yue Y, Kaur A, Lilja A, Diamond DJ, Walter MR, Barry PA. The susceptibility of primary cultured rhesus macaque kidney epithelial cells to rhesus cytomegalovirus strains. The Journal of general virology. 2016;97(6):1426-38. Epub 2016/03/15. doi: 10.1099/jgv.0.000455. PubMed PMID: 26974598; PubMed Central PMCID: PMCPMC5042092.

4.	Lockridge KM, Sequar G, Zhou SS, Yue Y, Mandell CP, Barry PA. Pathogenesis of experimental rhesus cytomegalovirus infection. J Virol. 1999;73(11):9576-83. Epub 1999/10/09. doi: 10.1128/jvi.73.11.9576-9583.1999. PubMed PMID: 10516066; PubMed Central PMCID: PMCPMC112992.
